# Splenic Embolism in Infective Endocarditis: A Systematic Review of the Literature with an Emphasis on Radiological and Histopathological Diagnoses

**DOI:** 10.3390/tropicalmed9040083

**Published:** 2024-04-12

**Authors:** Gabriel Santiago Moreira, Nícolas de Albuquerque Pereira Feijóo, Isabella Braga Tinoco-da-Silva, Cyntia Mendes Aguiar, Francijane Oliveira da Conceição, Gustavo Campos Monteiro de Castro, Mariana Giorgi Barroso de Carvalho, Thatyane Veloso de Paula Amaral de Almeida, Rafael Quaresma Garrido, Cristiane da Cruz Lamas

**Affiliations:** 1 Department of Medicine, Universidade do Grande Rio/Afya (UNIGRANRIO/Afya), Barra da Tijuca, Rio de Janeiro 22775-003, Rio de Janeiro, Brazil; gabrielsantiagom@live.com (G.S.M.); nicolasapfeijoo@gmail.com (N.d.A.P.F.);; 2 Instituto Nacional de Cardiologia, Rio de Janeiro 22240-006, Rio de Janeiro, Brazil; cy.aguiar@gmail.com (C.M.A.); franolive83@yahoo.com.br (F.O.d.C.); garrido.dip@gmail.com (R.Q.G.); 3 Instituto Nacional de Infectologia Evandro Chagas, Fiocruz, Rio de Janeiro 21040-360, Rio de Janeiro, Brazil

**Keywords:** computed tomography, embolism, endocarditis, histopathology, imaging, pathology, positron emission tomography, spleen, splenic emboli, tomography

## Abstract

Infective endocarditis (IE) is characterised by fever, heart murmurs, and emboli. Splenic emboli are frequent in left-sided IE. A systematic review of the literature published on splenic embolism (SE) between 2000 and 2023 was conducted. Search strategies in electronic databases identified 2751 studies published between 1 January 2000 and 4 October 2023, of which 29 were finally included. The results showed that the imaging tests predominantly used to detect embolisms were computed tomography (CT), magnetic resonance imaging, positron emission tomography (PET)/CT, single-photon emission computed tomography/CT, ultrasound, and contrast-enhanced ultrasound. More recent studies typically used ^18^F-FDG PET-CT. The proportion of SE ranged from 1.4% to 71.7%. Only seven studies performed systematic conventional CT screening for intra-abdominal emboli, and the weighted mean frequency of SE was 22% (range: 8–34.8%). ^18^F-FDG PET-CT was performed systematically in seven studies, and splenic uptake was found in a weighted mean of 4.5%. There was a lack of uniformity in the published literature regarding the frequency and management of splenic embolisation. CT scans were the most frequently used method, until recently, when ^18^F-FDG PET-CT scans began to predominate. More data are necessary regarding the frequency of SE, especially focusing on their impact on IE management and prognosis.

## 1. Introduction

Infective endocarditis remains a deadly disease, with an approximately 20% mortality rate, despite optimal medical and surgical treatment. It has a growing incidence related to ageing populations continuously exposed to healthcare, including hospitalisation and invasive procedures such as intravenous lines and haemodialysis [1,2,3,4]. It is caused by an endocardial infection, particularly of the surface of the heart valves, and the lining of the ventricles and atria. Its most frequent pathological manifestations are sessile vegetations [1,2,5,6] that can fragment and generate septic emboli, which can produce remote ischaemia and metastatic infection. In left-sided IE, this occurs in approximately 20–50% of cases, with the most commonly affected sites being the central nervous system and spleen. The risk factors described for the occurrence of embolisation were vegetation size greater than 10 mm, vegetation mobility, previous embolisms, multivalvular IE, mitral valve involvement, and a causative infectious agent (most commonly *S. aureus*, streptococci from the *bovis* group, and *Candida* spp.). The risk of new embolisms drops sharply after two weeks of antibiotic therapy, which reinforces the need for early diagnosis and treatment [5,7].

The main diagnostic features of IE are fever, new murmurs, and embolic phenomena. When IE affects the left-sided valves (mitral and/or aortic), systemic emboli occur more frequently in the intra-abdominal solid organs, central nervous system, lumbar spine, and skin. The spleen is the most commonly affected solid intrabdominal organ [1,2,3,4]. As highlighted in the European guidelines published in 2015, the imaging of embolic lesions is important. With the imaging of embolic lesions, minor criteria have been proposed, contributing to the definitive diagnosis of IE, especially in patients where IE is a very likely diagnosis [5].

Several imaging techniques are used for this purpose. Abdominal ultrasound (US) is a quick, inexpensive, bedside, and non-invasive examination in which splenic infarction is described as a triangular hypoechoic lesion with a base facing the periphery and well-defined borders, similar to the tomographic description. On Doppler scans, a reduction in local perfusion is observed; however, the presence of flow does not exclude the diagnosis of embolism because there is an evolutionary tendency towards the destruction of the embolus, re-establishing local flow due to a drop in vascular resistance [8,9,10]. When fibrosis is induced, a nodular image with smaller proportions is observed. Abscesses are described as focal images of varying echogenicity (anechoic, hypoechoic, or mixed), oval or fusiform, with an irregular wall, which may present with air–fluid levels and septations in addition to mild-to-moderate posterior acoustic enhancement. Hyperechogenic foci may be observed in patients with gas within an abscess. Furthermore, Doppler imaging reveals avascular patterns. However, since the sensitivity of lesion detection depends on the examiner, US is less precise than other examinations [8,9,10,11,12]. Contrarily, contrast-enhanced US (CEUS) uses a contrast medium that exhibits high uptake by splenic macrophages and does not disperse through the interstitial space, resulting in no contrast enhancement in infarcts and abscesses. This technique allows for a significant increase in ultrasound diagnostic gain, making reports more uniform and reproducible, in addition to increasing the sensitivity and specificity to values similar to those of CT and MRI. Therefore, this presents a promising option for those who cannot be exposed to radiation, such as pregnant women [10,13].

**Figure 1:**
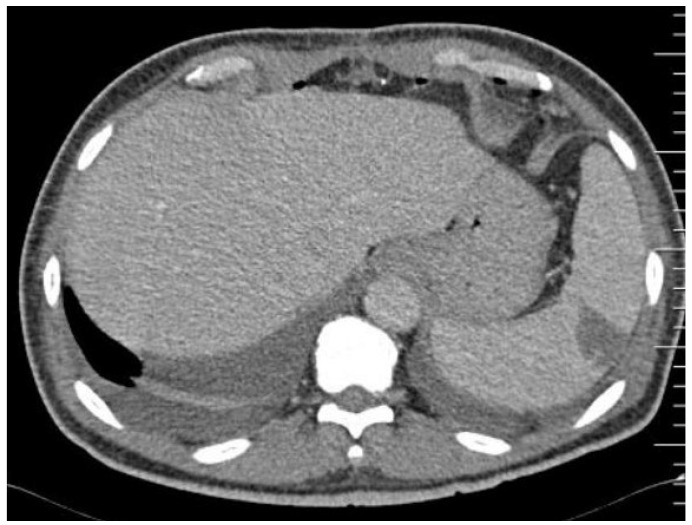
Peripheral splenic embolus on non-contrasted CT scan, wedge-shaped, subcapsular, from a patient with bicuspid aortic valve endocarditis caused by *Abiotrophia defectiva*. Source: Instituto Nacional de Cardiologia image collection, 2017.

In non-contrast CT, the normal splenic parenchyma is described as having a homogeneous pattern with an intermediate attenuation coefficient of approximately 40–60 Hounsfield units (HU). One minute after contrast administration, heterogeneous contrast uptake may be observed in the arterial and initial portal phases, with subsequent homogenisation in the venous phase [9,10,11,14,15,16,17,18]. In contrast, splenic infarcts are triangular hypodense lesions of varying size with a base facing the periphery (wedge-shaped), with a predominantly peripheral location, and without contrast enhancement (Figure 1).

**Figure 2:**
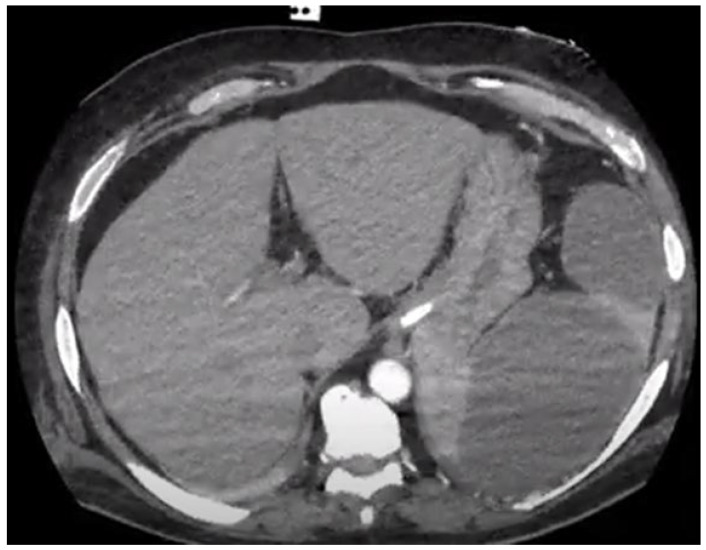
Large splenic abscesses in a patient on chronic haemodialysis with *Enterococcus faecalis* aortic valve endocarditis on non-contrasted CT scan of the abdomen. Source: Instituto Nacional de Cardiologia image collection, 2023.

In the hyperacute phase, hyperdense areas are interspersed with infarctions corresponding to small haemorrhagic foci. Subsequently, they become smaller, fibrotic, and denser, rarely progressing to abscesses (approximately 5% of cases). When the splenic artery is obstructed, infarcts can affect the entire spleen, which may result in splenic rupture with subcapsular and intraperitoneal haemorrhages, the latter of which carries the potential risk of haemorrhagic shock. Haematomas present as hyperdense lesions (60–80 HUs) without contrast uptake [9,10,11,14,15,16,17,18]. Splenic infarcts and abscesses may be considered different evolutionary phases of embolism formation because abscesses may result from a septic embolus or an aseptic infarction, making their radiological distinction difficult in the early stages. Splenic abscesses are described as hypodense lesions (20–40 HUs) of a central location with necrotic and fluid-filled centres (Figure 2). 

Classical peripheral contrast enhancement occurs when a capsule is formed. The presence of gas is rare and provides greater diagnostic accuracy. Fungal infections tend to generate multiple small lesions (up to 2 cm) that are poorly enhanced by contrast, similar to those caused by mycobacteria, which can lead to underdiagnosis. Monitoring the evolution of tomographic images can elucidate the difference between the two types of lesions; however, this may delay diagnosis and management and increase the risk of unfavourable outcomes [9,10,11,14,15,16,17,18].

MRI is a valuable tool for identifying intra-abdominal embolisms, with less toxicity and greater sensitivity than conventional CT; however, it has the disadvantage of causing discomfort to patients owing to the duration of the examination. On T1 weighting, the normal splenic parenchyma had a lower intensity than the liver and a greater intensity than the skeletal muscles, whereas, on T2 weighting, it was hyperintense compared to the liver. The signal intensity of infarcts varies according to their age; therefore, recent lesions are hyperintense on T1 weighting and subacute or chronic lesions are hypointense. Abscesses are characterised as fluid lesions with low intensity on T1 weighting and high intensity on T2, with possible peripheral contrast uptake by the capsule and/or perilesional reactive inflammation [9,10,12,18].

On ^18^F-FDG PET/CT imaging, a greater uptake of ^18^F-FDG is observed in areas affected by emboli. However, there are limitations to this technique, including difficulties in locating septic emboli in tissues with high physiological uptake, in hyperglycaemic states of critically ill patients where possible competition occurs between the marker and glucose, and in metastatic infections < 5 mm in size, which is below the spatial resolution limit of current scanners. The TEPvENDO study included 129 individuals who underwent whole-body ^18^F-FDG PET/CT in eight French hospitals. Diffuse splenic hypermetabolism was observed in 73% of the entire cohort, 82% of those with definitive IE, and 41% of those in whom a diagnosis of IE was rejected, confirming both the diagnostic potential of the examination and its potential for yielding ambiguous results [5,19,20].

At present, due to its availability and good image resolution, contrast abdominal CT is the examination of choice for the screening for embolisms, with the interpretation of subsequent scans being important in management. If images show that the condition is worsening or clinical features of fever and abdominal pain occur, these indicate a probable splenic abscess, and conservative treatment with antibiotics or percutaneous drainage or surgery, with splenectomy, may be recommended. However, the use of contrast must be carefully considered, given the risk of acute kidney injury due to nephropathy induced by iodinated contrast, in addition to renal dysfunction related to prolonged antibiotic therapy and complications intrinsic to the disease, such as acute diffuse glomerulonephritis due to the deposition of immune complexes, sepsis, and cardiac dysfunction [5,11,16,18,21,22].

Given the frequency of splenic embolisms, management thereof is important; however, there is still no consensus in the literature regarding the therapy of choice. There are three basic approaches: conservative therapy alone with antibiotics, antibiotics and percutaneous drainage, or surgical therapy with splenectomy. If no clear diagnosis of splenic abscess is made, the advised management strategy is intravenous antimicrobials for IE with follow-up CT scans while monitoring the clinical response. However, splenectomy should be considered in cases of imminent splenic rupture, such as large infarcts or abscesses > 200 cm that respond poorly to antibiotic therapy alone [5,11,23,24]. Percutaneous drainage is more likely to be successful when performed under the following conditions: unilocular or bilocular abscess collection, smooth wall without internal septations, the content is sufficiently liquid to be drained, and there are up to two collections located peripherally or in the middle and lower poles of the spleen. Multilocular abscesses with thick septations or necrotic debris respond poorly to percutaneous drainage [25]. Furthermore, complications, such as damage to the colon, stomach, left kidney, and pancreas, may occur because of the anatomical location of the spleen. The most common complication is bleeding, which can be diagnosed through post-procedural imaging studies and usually requires no intervention. However, some patients may require emergency splenectomy because of haemodynamic instability or, in some cases, after an unsuccessful drainage procedure [26,27].

According to the most recent guidelines, splenectomy should ideally be performed before valve surgery to avoid possible contamination of the prosthetic valve by de novo bacteraemia originating from the spleen. If heart surgery is not urgent, the two surgeries may be performed at different times [11,28]. Although possible, valve surgery and splenectomy are rarely performed simultaneously due to the increase in surgical morbidity and the need for two separate surgical teams (cardiac surgery and general surgery). Postoperatively, patients who underwent both surgeries (heart valve surgery and splenectomy at the same time) required a longer duration of mechanical ventilation [5,11,23].

The objective of this study was to conduct a systematic review of the literature on splenic embolism in the context of infective endocarditis, with an emphasis on the diagnostic methods used and the histopathological findings. Our specific objectives were to (i) identify the main clinical and epidemiological characteristics of patients with splenic embolism in IE, (ii) describe the radiological methods used to detect splenic embolism, and (iii) describe the histopathological findings of splenic embolism in IE.

## 2. Materials and Methods

This study was a systematic literature review of the literature that followed the Preferred Reporting Items for Systematic Reviews and Meta-Analyses (PRISMA) guidelines [29]. This review was registered in PROSPERO under the number CRD42021257353.

The search for available literature took place on the following platforms: Embase, PubMed, Bireme, and Scielo. The keywords selected for the search were: “Endocarditis”, “Spleen”, “Splenic emboli”, “Splenic embolism”, “Embolism”, “Tomography”, “Imaging”, “Pathology”, “Histopathology”, “Positron Emission Tomography”, and “Computed Tomography” and their equivalents in Portuguese, which were used as Medical Subject Headings (MeSHs) and Health Sciences Descriptors (DeCSs). Two search strategies were used based on either a combination of terms in each group or between groups of words using the Boolean operators “OR” and “AND”, respectively (Supplementary Materials, Box S1).

Strategy 1 sought information about endocarditis and splenic embolism using radiological criteria, whereas Strategy 2 focused on gathering literature on pathological data on IE and the spleen. Other publications were included through manual selection of the bibliographic references of the articles selected according to the inclusion criteria. Furthermore, additional relevant publications on IE were identified through the PubMed platform for the theoretical basis of the study (introduction and discussion).

The search data for the articles were extracted and organised into Excel spreadsheets with the following information: title, authors, year and volume of publication, journal title, and language. The inclusion criteria were as follows: age of participants >18 years, studies published in the last 24 years (from 1 January 2000 to 9 March 2021 and from 10 March 2021 to 4 October 2023), and publications in English, Spanish, or Portuguese. The exclusion criteria were non-systematic reviews of the literature, case reports, publications focusing on non-splenic embolism, publications in conference proceedings, and publications without reports of splenic embolism.

Eligibility was assessed by two independent reviewers (GSM and IBTS) in two stages. First, publications duplicated between search strategies were excluded, and only articles with titles close to the objectives of the present work were selected. An article was considered eligible if it was selected by at least one reviewer. In the second phase, the corresponding abstracts were read, those that met the predefined exclusion criteria were excluded, and the remaining articles were read in full to confirm their eligibility. Disagreements were resolved by consensus, and a third reviewer (CCL) was consulted when consensus was not possible. This process was repeated to include articles from 10 March 2021 to 4 October 2023, using the same search strategies, by two new reviewers, NF and GC; disagreements were resolved by consensus, and a third reviewer (CCL) was consulted when consensus was not possible.

## 3. Results

**Figure 3:**
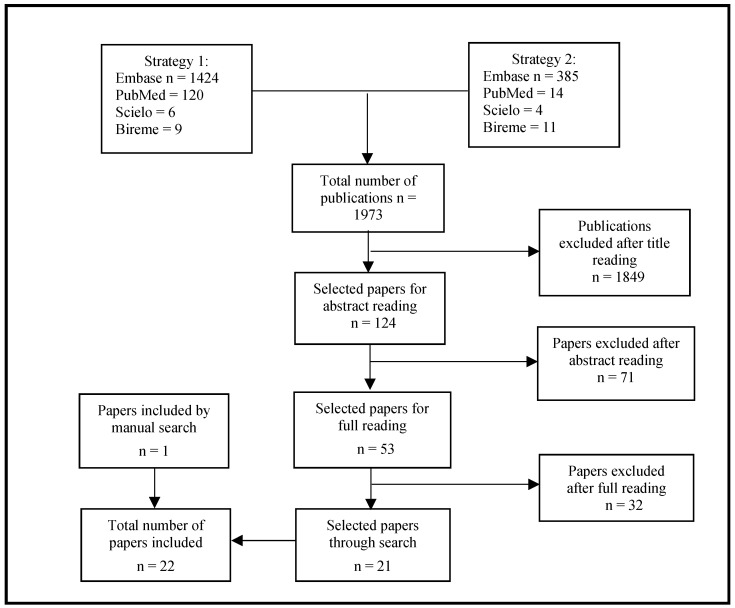
Flowchart for selecting publications based on search strategies 1 and 2; years 2000–2021.

The search strategies identified a total of 1973 articles published from 1 January 2000 to 9 March 2021, of which 1849 were excluded based on the title and 71 were excluded based on the abstract. After reading the remaining publications in full, a further 32 articles were excluded, and 21 eligible articles were finally identified. Only one publication was included from the manual search of the bibliographic references of the articles selected in the second phase; thus, a total of 22 articles were included in this review. Figure 3 presents a flowchart of the selection process based on the PRISMA methodology for the years 2000–2021.

**Figure 4:**
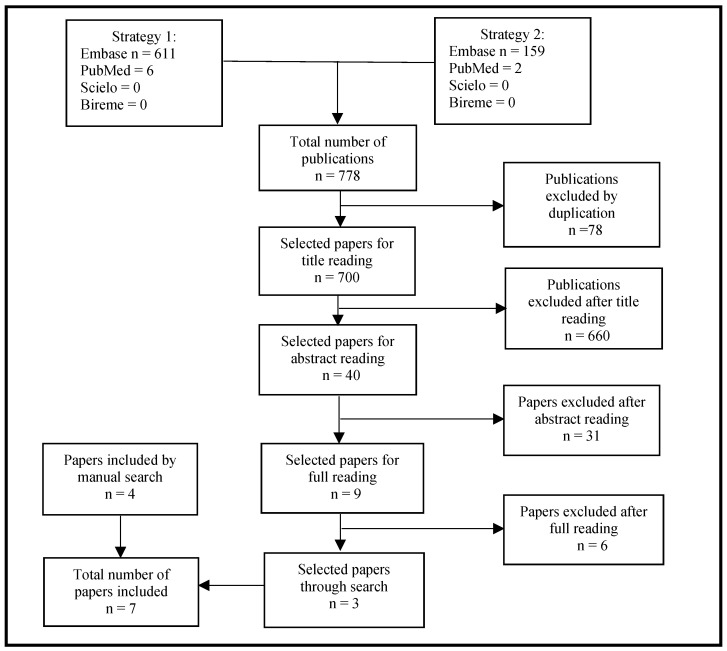
Flowchart for selecting publications based on search strategies 1 and 2; years 2021–2023.

Upon updating the literature search to include articles from 10 March 2021 to 4 October 2023, an additional 778 articles were found, of which 78 were excluded as they were duplicates. Of the 700 remaining articles, 660 were excluded after reading the title; 40 abstracts were read, after which a further 31 articles were excluded. After reading the remaining nine articles in full, six were excluded, and three were included. Four additional articles were included after a manual search, totalling seven in the updated search using strategy 1. No articles were selected using strategy 2. Figure 4 presents a flowchart of the selection process based on the PRISMA methodology for the years 2021–2023.

Table 1 presents the results obtained from strategy 1 (imaging examinations) over the entire literature search period and includes 27 articles [7,11,20,21,25,30,31,32,33,34,35,36,37,38,39,40,41,42,43,44,45,46,47,48,49,50,51]. Table 2 presents the results obtained from strategy 2 (pathology and histopathology), also for the whole period [52,53].

The number of left-sided IE episodes ranged from six to nearly three thousand patients [30,37], and the mean age of the patients ranged from 43 to 70 years. Males were the most affected in all studies (54–92.3%), except in the study by Menozzi et al., where only 47.6% were men; however, the very small number of patients included in that study must be considered (n = 6).

The imaging tests predominantly used to detect embolisms were USG [11], CT [7,11,21,25,30,31,32,33,34,41,43,45,46,50], MRI [43,51], ^18^F-FDG PET/CT [20,29,35,38,39,40,42,44,47,48,49,50,51], SPECT/CT [29,36], and CEUS [37]. More recent studies tended to use PET-CT, although many still used conventional CT as it is more readily available.

The percentage of cases with splenic embolisms ranged from 1.4% to 71.7%, which may be explained by the lack of systematic screening for intra-abdominal embolisms by considering only symptomatic patients [31,44] and by some studies that included patients with only possible IE or those that did not discriminate how many embolisms occurred in each group (left-sided or right-sided IE) [20,38,44].

Regarding the histopathological data presented in Table 2 [52,53], post-mortem spleen evaluation was performed in all patients; one study included 12 patients, and the other, 68. Splenic embolism occurred at different frequencies in these studies (12% and 39.7%, respectively). The histopathology of the spleen was only described in one article, demonstrating the frequency of infarcts as 32.3% and that of abscesses as 16.6% [52].

## 4. Discussion

Being predominant between the second and fourth weeks of the disease and less frequent after the start of antibiotic therapy, embolic phenomena may occur because they are intrinsic to the pathophysiology of IE [2,5,7,21,34,54]. We searched the literature published in the past two decades for inclusion in a systematic review of endocarditis and splenic emboli to provide a current and comprehensive overview of the radiological and histopathological findings on the subject.

We found that only seven (out of 27 selected manuscripts on radiological studies of splenic emboli) performed systematic tomographic screening for intrabdominal emboli in left-sided endocarditis, and the weighted mean frequency of splenic emboli was 22% (range: 8–34.6%) [21,25,32,34,45,46,50]. All studies were European, except for one conducted by our group in Brazil [25]. Conventional contrast-enhanced CT images may not differentiate between infarcts and abscesses except if done sequentially [9,10,11,14,15,16,17,18]. ^18^F-FDG PET/CT was performed systematically in seven studies, and they found splenic uptake in 1.4–24%, with a weighted mean of 4.5%. ^18^F-FDG PET/CT scan results do not usually report the presence of images with no uptake, although it is technically feasible as it involves plain CT images. The study using ^18^F-FDG PET/CT with the highest frequency of splenic emboli (24%) included only 25 patients, and the patients had the most advanced mean age in this report [35]. Notably, overall, the number of patients assessed using ^18^F-FDG PET/CT was much smaller than that of those assessed using conventional CT alone. Interestingly, one study did not describe focal uptake, but rather diffuse hypermetabolism, in 71% of patients [20]. They subsequently proposed that spleen hypermetabolism should be included as a minor diagnostic criterion for IE as it was a predictor of definite IE, independent of cardiac uptake. Although outside the scope of this review, bone marrow hypermetabolism was present in 59% of definite IE cases, and both spleen and bone marrow hypermetabolism occurred in 82% of patients with definite IE [20]. This underscores the importance of evaluating the spleen in endocarditis.

The frequency of splenic emboli (in addition to emboli at any other site) detection depends on the radiological method used (US scan, CT scan, MRI, PET/CT, SPECT/CT, etc.) and whether screening for emboli is requested systematically, requested at the discretion of the caregiver, or guided by patients’ symptoms and signs. ^18^F-FDG PET/CT scans usually report focal (and, less frequently, diffuse) splenic uptake and not infarcts (normal or hypocaptating lesions), even though CT images can show infarcts. Focal splenic uptake corresponds to an abscess, which is more specific to IE (and was therefore been included in the Duke-ISCVID criteria published in 2023) [55], and has been reported in less than 10% of cases. The standardisation of ^18^F-FDG PET/CT, as well as the way in which it is reported, needs to be improved in the future [56].

Most papers evaluated patients with IE with and without embolic events; no specific analysis regarding mortality and microbiology in patients with splenic emboli was performed except in a few [21,25,31]. One of them described, for the 46 patients with splenic embolism, that the main causative agents were *Staphylococcus aureus* (n = 17, 36.95%), viridans-group streptococci (n = 12, 26.08%), and *Enterococcus faecalis* (n = 9, 19.56%) [31]. They compared other features in those who only had splenic emboli (N = 17), only brain emboli (N = 13), and emboli to the spleen and brain (N = 16); numbers were small and there were no statistical differences between these three groups except for peripheral vascular disease, which was more frequent in those with splenic emboli only [31]. Moreover, an analysis for mortality was performed: in-hospital mortality occurred in four (8.69%) patients, all in the splenic emboli group, and three of these patients had a splenectomy. Noteworthily, in-hospital mortality was associated with splenic emboli (OR = 1.31, 95% CI: 1.0–1.7, *p* = 0.015), age > 35 years, and congestive heart failure [31]. Monteiro et al., in a publication by our group in 2017, had a special interest in the spleen [25]. We found in a sample of 136 adult patients with IE that mortality was 15.7% in patients who had cardiac surgery and 28.5% in those who did not (with no statistical difference). Embolism to the CNS or spleen, symptomatic or asymptomatic, and cardiac surgery or splenectomy did not impact mortality. We also investigated factors associated with embolism to the spleen, and these were splenomegaly (*p* < 0.01, OR = 9.28, 95% CI: 3.32–29) and positive blood cultures (*p* = 0.05, OR = 8.94, 95% CI: 1.45–177) [25]. Lastly, Parra and colleagues analysed differences between patients with IE who had combined spleen, liver, and kidney emboli [21]. They concluded that the site of acquisition, clinical characteristics, microbiology, surgical treatment, days of hospitalisation, hospital death, and 1-year mortality were similar in patients with and without splenic, renal, and liver emboli on CT [21].

Our study has several limitations. First, the publications on endocarditis and splenic embolism often did not state whether IE was left- or right-sided. Second, it was not clear in many of the papers whether imaging was performed systematically, symptom-orientated, or performed at the discretion of the attending physicians. Third, most of the studies were European and, therefore, generalisation to other populations may not be possible. Fourth, the frequencies of infarcts or abscesses were not clearly stated for many of the included manuscripts. Fifth, the impact of the splenic findings was not discussed. Lastly, although we recognise these many limitations, and believe the risk of bias was high for all studies, a formal assessment of the risk of bias was not performed. The strength of our study is that it reviews the recent literature on splenic emboli in IE, which may be an important finding in patients undergoing open-heart surgery. Furthermore, diffuse splenic hypermetabolism has been described as an important diagnostic finding in IE.

We conclude that there is a lack of uniformity in the published literature regarding the frequency and management of splenic emboli, which is the most common site of emboli in left-sided IE. Therefore, the purpose of the present study was to fill this gap in the literature and initiate debate on the real impact of splenic emboli as well as the best diagnostic and therapeutic approaches in such cases.

## 5. Conclusions

The radiological methods used to detect splenic embolisms were US, US with microbubble contrast, CT, MRI, ^18^F-FDG PET/CT, and SPECT/CT, with CT being the most widely available technique of choice in most studies, followed by ^18^F-FDG PET/CT, which was predominantly used in recent studies.

Splenic embolism was detected in studies where conventional CT scanning was systematically performed, with a weighted mean frequency of 22%. A weighted mean of 4.5% of splenic ^18^F-FDG uptake was found in those studies where ^18^F-FDG PET/CT scans were systematically performed.

## Figures and Tables

**Table 1:** Results of the systematic literature search, 2000–2023, on imaging of splenic embolism in infective endocarditis.

Author, Year, Country	Number of Episodes of Left-Sided IE	Splenic Emboli n (%)	Radiological Examination Method Used	Emboli to the CNS n (%)	Cardiac Surgery for IE n (%)	In-Hospital or 30-Day Mortality (%)
Di Salvo et al., 2001, France [32]	174	14/174 (8%)	CT Performed routinely for 167/178 (93.8%) patients	27/174 (15.5%)	109/178 (61%)	19/178 (10.7%)
Vilacosta et al., 2002, Argentina and France [7]	217 91% definite IE	6/34 (18%)	CT Not routinely performed	52%	115/217 (53%)	42.9% of those with emboli; 30.2% of those without emboli
Deprele et al., 2004, France [33]	80	27%	CT Not clear if performed systematically	34%	30/80 (37.5%)	7/80 (8.8%)
Thuny et al. 2005, France and Italy [34]	350	49/350 (14%)	CT Systematically performed at study entry	62/350 (17.7%)	52.3%	37/350 (9.6%)
Luaces Méndez et al., 2004, Spain [11]	338	34/338 (10%); 4/34 (11.8%) splenic abscess	US 30/34 (88.2%) or CT 26/34 (67.6%) Guided by signs/symptoms	77/338 (22.7%); 18/34 (52.9%)	181/338 (53.5%)	107/338 (31.6%)
Van Riet et al., 2010, Belgium [35]	25	6/25 (24%)	^18^F-FDG PET/CT Performed systematically 2 weeks after IE diagnosis	NA	17/25 (68%)	1/25 (4%)
Erba et al., 2012, Italy [36]	51	4/51 (7.8%)	SPECT/CT Performed in all patients	NA	NA	NA
Menozzi et al., 2013, Italy [37]	6	5/6 (83.3%)	CEUS Performed in all patients within 10 days after IE diagnosis	NA	NA	NA
Bonfiglioli et al., 2013, Italy [38]	29/71 unclear if left-sided or right-sided IE	1/17 (5.9%)	^18^F-FDG PET/CT Performed systematically	NA	NA	NA
Kestler et al., 2014, Spain [39]	38/47	3/47 (6.4%)	^18^F-FDG PET/CT Performed systematically	3/47 (6.4%)	30/47 (63.8%)	NA
Asmar et al., 2014, Denmark [40]	72 (majority left-sided IE)	1/72 (1.4%) abscess	PET/CT Performed systematically	NA	44%	15%
Rizzi et al., 2014, Italy [41]	1456 − (89 + 61) = 1306 (definite and possible)	113/1306 (8.6%)	CT Not performed systematically	242/1306 (18.5%)	NA	NA
Salomäki et al., 2015, Finland [42]	11/12	1/12 (8.3%)	^18^F-FDG PET/CT Performed systematically	NA	5/12 (41.7%)	1/12 (8.3%)
Aalaei-andalabi et al., 2017, United States of America [31]	437 surgical IE; 46 studied for emboli	33/46 (71.7%)	CT Guided by signs/symptoms	29/46 (63%)	100%	8.7%
Monteiro et al., 2017, Brazil [25]	119/136 (87.5%)	44/136 (32.8%)	CT All patients	32/136 (23.5%)	98/136 (72%)	24%
Takahashi et al., 2017, Japan [43]	166	5/166 (3%) “new emboli”	CT or MRI All patients	28/166 (17%)	87/166 (52%)	19%
Kouijzer et al., 2018, Netherlands [44]	10/88 (not specified if left-sided or right-sided)	7.9% splenic abscesses (definite and possible IE)	^18^F-FDG PET/CT All patients	NA	NA	NA
Parra et al., 2018, Spain [21]	147	44/147 (29.9%)	CT All included patients; 1/3 due to symptoms	37/147 (25.1%)	72/147 (48.9%)	34/147 (23.1%)
Selton-Suty et al., 2018, France [45]	133	46/133 (34.6%)	CT Routinely performed for all patients, but 57 were symptomatic	52/133 (39%)	89/186 (48%)	29/186 (16%)
Lecomte et al., 2019, France [46]	477/522 (91.4%)	131/522 (25.1%)	CT (thoraco–abdominal–pelvic) All patients	NA	NA	82/522 (15.8%) overall; 65/316 (20.6%) with emboli
Habib et al., 2019, multicentre, predominantly European [30]	3116 (308 were device-related)	10.1% overall; 22.3% of embolic events on admission	CT, ^18^F-FDG PET/ CT SPECT/CT All as per centre	350/788 (44.4%)	1596/3116 (51.2%)	17.1%
Boursier et al., 2019, France [20]	88/129	62/88 (71%) diffuse splenic hypermetabolism	^18^F-FDG PET/CT Performed systematically	NA	NA	NA
San et al., 2019, France [47]	173	24/173 (13.8%)	^18^F-FDG PET/CT Systematic	NA	93/173 (54%)	14/173 (8%)
Holle et al., 2020, Denmark [48]	169/178 definite left-sided IE	11/169 (6.5%)	^18^F-FDG PET/CT Performed systematically	NA	71/178 (40%)	13/178 (7%)
Li et al., 2022, Germany [49]	201	21/215 (9.8%); 21/62 (33.8%) of those who had ^18^F-FDG PET/CT	^18^F-FDG PET/CT Performed preferably in PVE	77/215 (35.8%)	201/201 (100%)	32/215 (14.9%)
Radjabaly Mandjee et al., 2022, France [50]	1502 − 80 = 1422	325/1502 (21.63%)	MSCT in 1319 patients ^18^F-FDG PET/CT in 217 patients	552/1502 (36.8%)	53.5% and 36.3%	550/1488 (37%)
Ucciferri et al., 2022, Italy [51]	68	12/68 (17.6%)	MRI-^18^F-FDG PET/CT Not systematically performed	7/68 (10.3%)	NA	20.6%

IE, infective endocarditis; CNS, central nervous system; CT, computed tomography; MSCT = multislice computed tomography; US, ultrasonography; SPECT, single-photon emission computed tomography; MRI, magnetic resonance imaging; CEUS, contrast-enhanced ultrasonography; PET, positron emission tomography; NA, not available.

**Table 2:** Information on manuscripts obtained after the systematic literature review on splenic emboli, histopathology, and pathology in infective endocarditis (2000–2023).

Author, Year, Country	Number of Patients Studied, Type of Valve	Method of Analysis	Mean Age (Years)	Splenic Embolism n (%)	Splenic Findings n (%)	Emboli to the CNS n (%)
Fernández Guerrero et al., 2019, Spain [52]	68 40P1 28P2 60 left-sided IE	Autopsy	46.6—Period 1 57.6—Period 2	27/60 (45%)	Infarct: 22/27 (81.5%) Abscess: 5/27 (18.5%)	20/68 (29.4%)
Berlot et al., 2014, Italy [53]	12	Autopsy	66	4/12 (33.3%)	NA	5/12 (41.6%)

IE, infective endocarditis; P1, period 1; P2, period 2; CNS = central nervous system; NA = not available.

## Data Availability

The data presented in this study are available on request from the corresponding author. The data are not publicly available due to the large extension of files obtained in the literature search.

## Supplementary Materials

The following supporting information can be downloaded at https://www.mdpi.com/article/10.3390/tropicalmed9040083/s1, Box S1. Terms used for literature search on radiological and histopathological aspects of splenic emboli in infective endocarditis.
