# Supplementary material for: Splenic Embolism in Infective Endocarditis: A Systematic Review of the Literature with an Emphasis on Radiological and Histopathological Diagnoses
Source: Trop Med Infect Dis. 2024 Apr 12;9(4):83. doi: 10.3390/tropicalmed9040083 (PMC11053958; doi:10.3390/tropicalmed9040083)
Supplement: Supplementary file 1 [file tropicalmed-09-00083-s001.zip › tropicalmed-2909624-supplementary.pdf]

Box S1. Terms used for literature search on radiological and histopathological aspects of splenic emboli in infective endocarditis.

PUBMED 1	(("endocarditis"[MeSH Terms] OR "Endocarditides"[Title/Abstract] OR "Infective Endocarditis"[Title/Abstract] OR "endocarditis infective"[Title/Abstract]) AND ("spleen"[MeSH Terms] OR "splenic emboli"[Title/Abstract] OR "splenic embolism"[Title/Abstract] OR "embolism"[MeSH Terms] OR "Embolisms"[Title/Abstract] OR "Embolus"[Title/Abstract]) AND ((("endocarditis"[MeSH Terms] OR "Endocarditides"[Title/Abstract] OR "Infective Endocarditis"[Title/Abstract] OR "endocarditis infective"[Title/Abstract]) AND ("spleen"[MeSH Terms] OR "splenic emboli"[Title/Abstract] OR "splenic embolism"[Title/Abstract] OR "embolism"[MeSH Terms] OR "Embolisms"[Title/Abstract] OR "Embolus"[Title/Abstract]) AND ("tomography"[MeSH Terms] OR "Tomographies"[Title/Abstract] OR "Imaging"[Title/Abstract] OR "positron emission tomography computed tomography"[MeSH Terms] OR "pet ct scan"[Title/Abstract] OR "pet ct scans"[Title/Abstract] OR "scan pet ct"[Title/Abstract] OR "scans pet ct"[Title/Abstract] OR "pet ct scan"[Title/Abstract] OR "ct scan pet"[Title/Abstract] OR "ct scans pet"[Title/Abstract] OR "pet ct scans"[Title/Abstract] OR "scan pet ct"[Title/Abstract] OR "scans pet ct"[Title/Abstract] OR "CT PET"[Title/Abstract] OR "positron emission tomography computed tomography"[Title/Abstract] OR "PET-CT"[Title/Abstract] OR "CT PET Scan"[Title/Abstract] OR "CT PET Scans"[Title/Abstract] OR "pet scan ct"[Title/Abstract] OR "pet scans ct"[Title/Abstract])) OR "positron emission tomography"[MeSH Terms] OR "positron emission tomography"[Title/Abstract] OR "positron emission tomography imaging"[Title/Abstract] OR "imaging positron emission tomography"[Title/Abstract] OR "positron emission tomography imaging"[Title/Abstract] OR "tomography positron emission"[Title/Abstract] OR "tomography positron emission"[Title/Abstract] OR "PET Scan"[Title/Abstract] OR "PET Scans"[Title/Abstract] OR "scan pet"[Title/Abstract] OR "PET Imaging"[Title/Abstract] OR "imaging pet"[Title/Abstract] OR "PET Imagings"[Title/Abstract]))<br><br>Filters applied: Adult: 19+ years
PUBMED 2	(("endocarditis"[MeSH Terms] OR "Endocarditides"[Title/Abstract] OR "Infective Endocarditis"[Title/Abstract] OR "endocarditis infective"[Title/Abstract]) AND ("spleen"[MeSH Terms] OR "splenic emboli"[Title/Abstract]

	OR "splenic embolism"[Title/Abstract] OR "embolism"[MeSH Terms] OR "Embolisms"[Title/Abstract] OR "Embolus"[Title/Abstract]) AND (("Pathology"[MeSH Terms] OR "Pathology"[Title/Abstract] OR "Pathologies"[Title/Abstract] OR "histopathology"[Title/Abstract]) AND "adult"[MeSH Terms]) AND (2021/03/10:2023/10/03[Date - Publication] AND "adult"[MeSH Terms])) AND (alladult[Filter]))<br><br>Filters applied: Adult: 19+ years
EMBASE 1	('endocarditis'/exp OR 'endocardial inflammation' OR 'endocarditis' OR 'paraneoplastic endocarditis' OR 'parietal fibroplastic endocarditis') AND ('spleen'/exp OR 'spleen' OR 'splenic embolism' OR 'splenic emboli' OR 'embolism'/exp OR 'embolism' OR 'embolism recurrence' OR 'embolus') AND ('tomography'/exp OR 'elliptical tomography' OR 'laminagraphy' OR 'laminography' OR 'narrow angle zonography' OR 'planigraphy' OR 'planography' OR 'planygraphy' OR 'polytomography' OR 'radiotomography' OR 'tomography' OR 'transverse section imaging' OR 'zonography' OR 'imaging'/exp OR 'imaging' OR 'positron emission tomography'/exp OR 'pet scan' OR 'pet scanning' OR 'p.e.t.' OR 'positron emission tomographic scan' OR 'positron emission tomographic scanning' OR 'positron emission tomography' OR 'positron tomography' OR 'positron-emission tomography' OR 'tomography, positron' OR 'positron emission tomography-computed tomography'/exp OR 'computer assisted positron emission tomography' OR 'positron emission computed tomography' OR 'positron emission tomography computed tomography' OR 'positron emission tomography, computer assisted' OR 'positron emission tomography-computed tomography' OR 'positron-emission tomography and computed tomography') AND ([adult]/lim OR [aged]/lim)
EMBASE 2	('endocarditis'/exp OR 'endocardial inflammation' OR 'endocarditis' OR 'paraneoplastic endocarditis' OR 'parietal fibroplastic endocarditis') AND ('spleen'/exp OR 'spleen' OR 'splenic embolism' OR 'splenic emboli' OR 'embolism'/exp OR 'embolism' OR 'embolism recurrence' OR 'embolus') AND ('pathology'/exp OR 'clinical pathology' OR 'pathobiology' OR 'pathologic institute' OR 'pathology' OR 'pathology institute' OR 'pathology, clinical' OR 'pathology, surgical' OR 'histopathology'/exp OR 'histopathological study' OR 'histopathology' OR 'neurohistopathology')
